# Engineered Phage Modulates Quorum Sensing and Biofilm Formation in *Pseudomonas aeruginosa*

**DOI:** 10.3390/microorganisms14051028

**Published:** 2026-04-30

**Authors:** Domenico Franco, Salvatore Papasergi, Francesco Mediati, Salvatore P. P. Guglielmino, Laura Maria De Plano

**Affiliations:** 1 Department of Chemical, Biological, Pharmaceutical and Environmental Sciences (ChiBioFarAm), University of Messina, Viale F. Stagno d’Alcontres 31, 98166 Messina, Italy; francesco.mediati@studenti.unime.it (F.M.); sguglielm@unime.it (S.P.P.G.); 2 Institute of Translational Pharmacology of the National Research Council, Via Fosso del Cavaliere, 100 (c/o Area di Ricerca di Tor Vergata), 00133 Rome, Italy; salvatore.papasergi@cnr.it; 3 Department of Occupational and Environmental Medicine, Epidemiology and Hygiene, Italian Workers’ Compensation Authority (INAIL), Contrada Ficarella, 88046 Lamezia Terme, Italy

**Keywords:** phage-display technology, *P. aeruginosa*, quorum sensing network, biofilm

## Abstract

*Pseudomonas aeruginosa* is an opportunistic Gram-negative pathogen frequently associated with chronic and biofilm-related infections, largely driven by quorum sensing (QS)-related genes/phenotypes. In this study, we investigated the antivirulence activity of an engineered M13-derived phage-display particle (P9b), selected for specific binding to *P. aeruginosa*, which acts as a non-lytic modulator of QS through specific binding to a bacterial surface target. P9b induced a transient delay in early planktonic growth, without affecting long-term proliferation. In contrast, P9b significantly reduced biofilm-associated metabolic activity and pyocyanin production, consistent with an effect on QS-regulated pathways. Transcriptional analysis revealed significant downregulation of key QS regulators (*lasI*, *lasR*, *rhlI*, and *rhlR*) and modulation of phenazine biosynthesis genes (*phzM* downregulation and *phzS* upregulation), suggesting interference with QS-dependent regulatory circuits. Notably, P9b retained binding capacity and antibiofilm activity across clinically relevant *P. aeruginosa* isolates. Overall, these findings indicate that P9b acts as a selective, non-lytic modulator of virulence-associated traits, attenuating QS-regulated phenotypes without bactericidal effects. This study supports the potential of engineered filamentous phages as targeted antivirulence platforms for the development of innovative strategies against persistent and biofilm-associated infections.

## 1. Introduction

*Pseudomonas aeruginosa* is a Gram-negative opportunistic pathogen responsible for a wide range of acute and chronic infections, particularly in immunocompromised individuals and patients with cystic fibrosis, burn wounds, or implanted medical devices [1,2,3]. Its remarkable ability to persist in hostile environments is largely attributed to intrinsic and acquired antibiotic resistance mechanisms, as well as to its capacity to form highly structured biofilms [4,5,6,7].

A key determinant of *P. aeruginosa* pathogenicity is its sophisticated hierarchical quorum sensing (QS) network, which orchestrates the coordinated expression of virulence factors, biofilm maturation, and metabolic adaptation in a cell–density–dependent manner [8,9,10,11]. Disruption of QS signaling can effectively attenuate pathogenicity without exerting strong selective pressure for resistance [12,13,14].

In this context, bacteriophage-derived platforms have emerged as promising tools for the targeted control of bacterial pathogens [15,16]. Specifically, filamentous phages and engineered phage particles may modulate bacterial physiology through non-lytic processes, interacting with surface-exposed structures [17]. Phage display technology enables the selection of peptides with high specificity for bacterial surface structures, allowing phage-derived clones to directly interact with outer membrane targets and modulate bacterial regulatory pathways without requiring phage replication or genetic modification [18,19].

Recent studies have highlighted that engineered filamentous phages can influence bacterial behavior by perturbing envelope integrity, altering signal transduction pathways, or interfering with QS-regulated networks, ultimately leading to reduced biofilm formation and virulence factor production [20]; however, these mechanisms remain not yet fully understood. In particular, whether phage display–selected filamentous phages can influence quorum sensing (QS) regulatory networks in a non-lytic manner through direct interaction with bacterial surface targets remains unclear, and evidence linking surface-binding phage clones to transcriptional reprogramming of QS systems is still limited.

Building on these advances, in the present study, we investigated the antivirulence potential of the engineered M13-derived phage-display particle, P9b, previously selected for its high specificity toward *P. aeruginosa*. Here, we show that a phage display–derived filamentous phage can influence QS-related gene expression and virulence-associated phenotypes through specific surface binding, without requiring phage replication. Using an integrated phenotypic and transcriptional approach, we assessed whether P9b may influence QS-regulated virulence through specific interactions with bacterial surface structures. Furthermore, we evaluated whether these effects are conserved across clinically relevant *P. aeruginosa* isolates, providing preliminary insight into the translational potential of phage display–derived antivirulence strategies. This study represents a preliminary in vitro evaluation conducted on a limited number of clinical *P. aeruginosa isolates*. Nevertheless, the consistency of the observed effects supports the potential of this approach and provides a solid basis for further investigation in more complex models and larger strain collections.

## 2. Materials and Methods

### 2.1. Bacteriophages

Phage clone P9b used in this study is an engineered M13-derived phage-display particle obtained from the selection of phage peptide library for specific binding to the cell surface of *Pseudomonas aeruginosa* ATCC 2785. The library (kindly provided by Prof. F. Felici) was constructed using the pC89 vector, designed for the expression of random peptides fused to the major coat protein pVIII. P9b displays the peptide sequence QRKLAAKLT and was previously evaluated for its ability to recognize a molecular target present on the surface of *P. aeruginosa* [21]. As a control, the pC89 vector (M13 wild-type vector lacking peptide insertion) was used to assess whether the observed effects were specifically attributable to the displayed foreign peptide. Propagation of both pC89 and P9b was performed using *Escherichia coli* TG1 (lacZ-deficient) as the bacterial host.

### 2.2. Phage Propagation

*Escherichia coli* TG1 strain (Kan^−^, Amp^−^, lacZ^−^) was cultured to mid-log phase (OD_600_ = 0.7) and subsequently infected with either pC89 or P9b (Amp^+^). The infection was performed at 37 °C under static conditions for 15 min, followed by incubation with shaking at 250 rpm for 20 min. After infection, suitable dilutions were plated onto Luria–Bertani (LB) agar plates (20 g/L agar) supplemented with ampicillin (50 µg/mL) and incubated at 37 °C under static conditions. A single transformed colony of *E. coli* TG1 was selected and inoculated into 10 mL of LB medium containing ampicillin (50 µg/mL), then grown at 37 °C with shaking (250 rpm) until reaching an OD_600_ of 0.2. At this point, isopropyl β-D-1-thiogalactopyranoside (IPTG; 40 µg/mL) and helper phage M13K07 (Kan^+^; 10^9^ transducing units per milliliter, TU/mL) were added. The culture was incubated statically at 37 °C for 30 min, followed by an additional 30 min under gentle agitation. Cells were collected by centrifugation at 8000× *g*, resuspended in 500 mL of LB medium containing ampicillin (50 µg/mL) and kanamycin (10 µg/mL), and incubated overnight at 37 °C with shaking. The culture was subsequently centrifuged at 8000× *g* for 20 min at 25 °C, and the supernatant was recovered and mixed with 25% (*v*/*v*) polyethylene glycol (PEG)/NaCl solution. After incubation on ice for 4 h, phage particles were precipitated by centrifugation at 15,000× *g* for 45 min at 4 °C. The pellet was resuspended in Tris-buffered saline (TBS; 10% *v*/*v*) and subjected to a 25% (*v*/*v*) PEG/NaCl, and incubated on ice for an additional 4 h, followed by centrifugation under the same conditions. The final pellet, containing phage particles, was resuspended in TBS (10% *v*/*v*), filtered through a 0.22 µm pore-size membrane, and stored at 4 °C until further use. Phage titers were determined by infecting susceptible *E. coli* TG1 cells and calculating transducing units per milliliter (TU/mL) based on colony formation on LB agar plates supplemented with ampicillin. Plates, containing between 30 and 300 colonies, were counted to derive the number of active viral particles able to infect the host cell and express the transgene. Thus, the active particles were determined according to the following equation:

$$\frac{TU}{mL}=\frac{Number\;of\;Colonies\;}{Volume\;(0.1\;mL)\;}\times Dilution\;Factor$$

### 2.3. Bacterial Pathogenic Targets

Pseudomonas aeruginosa ATCC 27853 was sourced from the American Type Culture Collection (LGC Promochem, Milan, Italy). Alongside this reference strain, two clinical isolates were included in the study: *P. aeruginosa* Pr, characterized by pyorubin production, and *P. aeruginosa* Pc, known for producing pyocyanin. These isolates were selected based on their distinct phenotypic features and antibiotic resistance profiles. Clinical isolates were identified by matrix-assisted laser desorption/ionization time-of-flight mass spectrometry (MALDI-TOF MS), following the manufacturer’s instructions (Bruker Daltonics, Milan, Italy). Further methodological details are reported in our previous study [22]. All experiments were performed in Luria–Bertani (LB) broth, which served as both the basal medium and the control condition.

### 2.4. Avidity ELISA Test for P9b Against Laboratory and Clinical P. aeruginosa Strains

Flat-bottom 96-well ELISA plates (NuncMulti-sorp) were coated overnight at 4 °C with 100 μL of bacterial suspensions (OD_540_ = 1) of *P. aeruginosa* ATCC 27853, and the clinical isolates (*P. aeruginosa* Pr and Pc) were fixed in carbonate coating buffer (35 mM NaHCO_3_, 15 mM Na_2_CO_3_, pH 9.6) [18,23,24]. After incubation, wells were washed three times with washing buffer (PBS containing 0.05% Tween 20) and subsequently blocked with blocking buffer (6% non-fat dry milk and 0.05% Tween 20 in PBS) for 2 h at 37 °C. Plates were then washed five additional times prior to incubation with 100 μL of P9b or control phage (10^12^ Transducing Units per milliliter, TU/mL) for 1 h at 37 °C under gentle shaking. Following incubation, wells were washed five times and then incubated with 100 μL of anti-M13 horseradish peroxidase (HRP)-conjugated antibody (Amersham Biosciences, Buckinghamshire, UK), diluted 1:2500 in blocking buffer (1% non-fat dry milk and 0.05% Tween 20 in PBS), for 1 h at 37 °C. After a further five washing steps, 100 μL of tetramethylbenzidine (TMB) substrate was added, and the reaction was allowed to proceed for 45 min at room temperature. The reaction was stopped by adding 100 μL of 1 M H_2_SO_4_, and optical density was measured at 450 nm using a microplate reader (PerkinElmer Wallac 1420 Victor2, Turku, Finland).

### 2.5. Bacterial Growth Assessment

Bacterial growth kinetics were evaluated by viable cell count determination over time. Briefly, *P. aeruginosa* strains were grown overnight in Luria–Bertani (LB) broth at 37 °C with agitation (180 rpm). Bacterial suspensions were adjusted to a turbidity corresponding to 0.5 McFarland standard and subsequently diluted in fresh LB medium to obtain a final concentration of approximately 10^7^ CFU/mL. Aliquots of 10 mL of the standardized suspensions were transferred into sterile flasks and added with either pC89 or P9b at a final concentration of 10^12^ TU/mL, corresponding to a multiplicity of infection (MOI) of approximately 10^5^. Control conditions included untreated bacteria. Bacterial cultures were incubated at 37 °C under constant agitation (180 rpm), and growth was evaluated over time by determining colony-forming units (CFU). During the initial 8 h, 100 μL aliquots were collected at hourly intervals, serially diluted in sterile phosphate-buffered saline (PBS), and plated in triplicate on LB agar plates. Additional time points were recorded at 24, 48, and 72 h. Following incubation at 37 °C for 16–18 h, colonies were counted and the results expressed as CFU/mL. In parallel, optical density measurements were taken at 540 nm (OD_540_) at the same time points to monitor culture turbidity.

### 2.6. Biofilm Assay

The capacity for biofilm formation was evaluated using flat-bottom 96-well polystyrene microtiter plates, following a modified version of the method described by Coffey and Anderson protocol [25]. Overnight cultures of *P. aeruginosa* ATCC 27853 and the clinical isolates (*P. aeruginosa* Pr and Pc) were standardized to 0.5 McFarland turbidity (approximately 10^8^ CFU/mL) to promote early quorum sensing (QS) activation and biofilm establishment. For each condition, 200 μL of each standardized suspension was dispensed into wells in triplicate and added with either pC89 or P9b at a final concentration of 10^12^ TU/mL, resulting in a multiplicity of infection (MOI) of approximately 10^4^. Plates were incubated under static conditions at 37 °C for 24, 48, and 72 h to monitor both early and mature stages of biofilm formation. After incubation, planktonic cells were removed, and well was gently rinsed three times with 300 μL of phosphate-buffered saline (PBS, pH 7.4). During each wash, PBS was added, the plate was subjected to mild agitation for 5 min, and the liquid was carefully aspirated. After the final wash, 200 μL of PBS and 50 μL of Cell Proliferation Kit II (XTT, SigmaAldrich, Milan, Italy) solution (prepared according to the manufacturer’s instructions) were added to each well. Plates were incubated in the dark at 37 °C for 3 h. Subsequently, optical density was recorded at 490 nm (OD_490_) using a microplate reader (PerkinElmer Wallac 1420 Victor2, Turku, Finland) after brief gentle mixing. Absorbance was measured at 490 nm (OD490) using a microplate reader (PerkinElmer Wallac 1420 Victor2, Turku, Finland) after brief mixing. Background values, obtained from wells containing sterile PBS and XTT treated under the same conditions, were subtracted from all readings. Biofilm reduction (Br) for each condition was expressed as a percentage relative to the LB control using the following formula:

$$Biofilm\;reduction\;(\%)=\left(\frac{A-B}{A}\right)\times 100$$

where *A* represents the absorbance of the untreated control (LB), and *B* corresponds to the absorbance measured in the tested conditions (cultures treated with pC89 or P9b).

### 2.7. Pyocyanin Assay

Pyocyanin production by *P. aeruginosa* strains was quantified under the same experimental conditions and incubation time points adopted for reduction in biofilm-associated metabolic activity assays, following the protocol described by Zanni et al. with minor modifications [26]. Briefly, 800 μL from each experimental condition were collected in sterile microcentrifuge tubes and centrifuged at 6000× *g* for 10 min to remove bacterial cells. The resulting supernatants were transferred to a 96-well microtiter plate and analyzed using a microplate reader (PerkinElmer Wallac 1420 Victor2, Turku, Finland). Optical density was measured at 405 nm, and values were corrected by subtracting the background signal obtained from fresh, uninoculated culture media. Pyocyanin reduction was calculated as a percentage relative to the LB control using the same calculation approach described for biofilm reduction.

### 2.8. RNA Isolation

Total RNA was isolated from bacterial biofilms using TRIzol^®^ Reagent (Thermo Fisher Scientific, Waltham, MA, USA), with slight adaptation to improve lysis of biofilm matrix [27]. Biofilms from all experimental conditions were carefully collected from the growth surface by gentle scraping during the early stationary phase (12–16 h), transferred into RNase-free microcentrifuge tubes, and centrifuged at 10,000× *g* for 5 min at 4 °C. The resulting pellet was resuspended in 1 mL of TRIzol and homogenized by vortexing for 5 min, followed by sonication for 2 min. Samples were incubated at room temperature for 15 min to ensure complete dissociation of nucleoprotein complexes. Subsequently, 200 µL of chloroform was added to each sample, vigorously mixed, and incubated for 3 min before centrifugation at 12,000× *g* for 15 min at 4 °C. The aqueous phase was carefully transferred to a fresh tube and mixed with an equal volume of 100% ethanol. After centrifuging at 12,000× *g* for 10 min, the RNA pellet was washed with 75% ethanol, centrifuged again, air-dried, and finally resuspended in RNase-free water. RNA concentration and purity were determined by spectrophotometry.

### 2.9. Reverse Transcription PCR (RT-PCR)

Complementary DNA (cDNA) was generated from total RNA using the ImProm-II™ Reverse Transcription System, in accordance with the manufacturer’s instructions. In brief, 1 µg of total RNA was used as input for each 20 µL reaction. The RNA sample was initially combined with 0.5 µg of Random Hexamers primers in a final volume of 5 µL, heated to 70 °C for 5 min, and then rapidly cooled on ice for at least 5 min to facilitate primer annealing. Following this step, 15 µL of the reverse transcription master mix was added to each tube. This mix includes reaction buffer, MgCl_2_ to a dNTP mix (10 mM each), and 1 µL of reverse transcriptase and 1 µL of RNasin^®^ Ribonuclease Inhibitor (40 U/µL) to protect RNA from degradation. The reverse transcription was performed in a thermal cycler under the following conditions: 25 °C for 5 min (primer extension initiation), 42 °C for 60 min, and 70 °C for 15 min. The synthesized cDNA was stored at –20 °C and later used as a template for quantitative PCR analysis.

### 2.10. Primer Design and Quantitative Real-Time PCR

mRNA sequences for *lasI*, *lasR*, *rhlI*, *rhlR*, *phzM*, and *phzS* were obtained from the NCBI Reference Sequence (RefSeq). Gene-specific primers were designed with the Primer-BLAST tool (NCBI, Bethesda, MD, USA) [28], following the approach previously reported [22]. The selected primer sequences were commercially obtained and subsequently used in real-time PCR (qRT-PCR) to confirm differential gene expression in analyzed samples. Expression analysis was performed using SsoAdvanced™ Universal SYBR^®^ Green Supermix (Bio-Rad, Hercules, CA, USA). Each 10 μL reaction contained 5 μL of SYBR Green Supermix, 0.4 μL each of forward and reverse primers, 2 μL of cDNA, and nuclease-free water. All reactions were carried out in triplicate using the following conditions: 95 °C for 2 min, followed by 40 cycles of 95 °C for 3 s and 60 °C for 30 s. No-template controls (NTCs) were included for every primer pair. Relative gene expression was determined using the 2^–ΔΔCt method [29]. Enzyme Δ1-pyrroline 5-carboxylate reductase involved in proline biosynthesis (*proC*) was used as the endogenous reference gene [30].

### 2.11. Statistical Analysis

All experiments were performed in three independent biological replicates, and data are presented as mean ± standard deviation (SD). Statistical analyses were carried out using GraphPad Prism (7.0) software (GraphPad Software, San Diego, CA, USA). Data were assumed to follow a normal distribution, and variance was considered comparable between groups. The choice of statistical test was based on the number of experimental groups and the study design. For comparisons involving more than two groups, statistical significance was assessed using one-way analysis of variance (ANOVA), followed by Tukey’s post hoc test for multiple comparisons. For comparisons between two groups, an unpaired Student’s *t*-test was applied. A *p*-value < 0.05 was considered statistically significant.

## 3. Results

### 3.1. Effect of P9b on P. aeruginosa Planktonic Growth Kinetics

**Figure 1:**
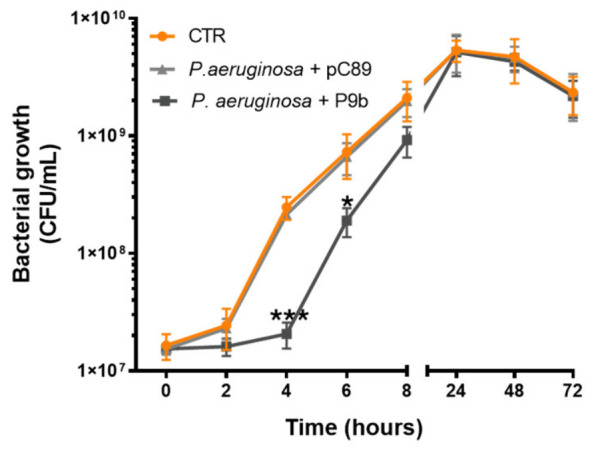
Growth kinetics of *Pseudomonas aeruginosa* ATCC 27853 alone or co-inoculated with the engineered M13-derived phage-display particle P9b or the insert-less vector pC89 (used as a control for phage-related effects not associated with the displayed insert). Data represent the means of three independent replicates. Statistical significance was evaluated using one-way ANOVA followed by Tukey’s post hoc test for multiple comparisons (Table S1). Adjusted *p*-values < 0.05, <0.01, and <0.001 are indicated by one (*), two (**), and three (***) asterisks, respectively.

To evaluate the effect of engineered M13-derived phage-display particle P9b on the growth of *Pseudomonas aeruginosa* ATCC 27853, growth curve analyses were performed under three conditions: bacteria alone (control for normal bacterial growth), bacteria co-inoculated with P9b, and bacteria co-inoculated with the pC89 (M13 wild-type vector lacking peptide insertion). Bacterial growth was monitored by viable cell counting at 0, 2, 4, 6, and 8 h, and subsequently at 24, 48, and 72 h (Figure 1, Table S1). The latter time points were selected to align with those used for biofilm inhibition assays, allowing direct comparison between planktonic growth and biofilm-associated phenotypes.

Growth curve analysis revealed that P9b induced a measurable but transient inhibition of *P. aeruginosa* growth. No differences were observed at the initial time point (0 h), indicating that phage exposure did not affect baseline cell density. A modest reduction in growth was already detectable at 2 h (*p* = 0.3051), becoming more pronounced at 4 h (*p* = 0.0004), corresponding to the phase in which P9b exerted its strongest inhibitory effect, visible as an extension of the lag phase. Phage clone effect was already maintained at 6 h (*p* = 0.0460) and tended to resolve by 8 h (*p* = 0.0919). This transient delay may reflect an early interaction between P9b and bacterial surface structures, leading to a temporary perturbation of growth dynamics. At later observation times (24, 48, and 72 h), no differences were detected between treated and untreated cultures, indicating that the inhibitory effect had largely dissipated and suggesting recovery of bacterial proliferation. In contrast to P9b, the insert-less vector pC89 did not induce any detectable changes in growth compared with the untreated *P. aeruginosa* control. Obtained results were also confirmed by spectrophotometric measurements (Figure S1, Table S2), although optical density readings were not fully informative beyond the early growth phase (after 8 h).

### 3.2. Effect of P9b on P. aeruginosa Biofilm Metabolic Activity

**Figure 2:**
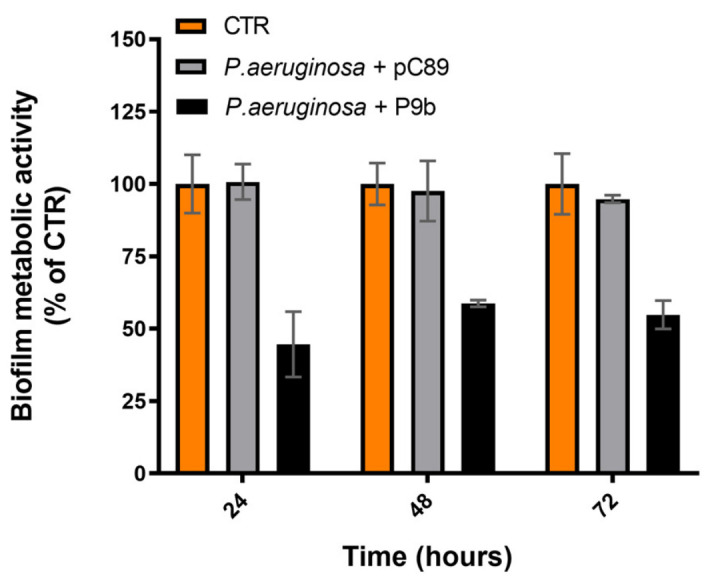
Biofilm metabolic activity (XTT, OD_490_) of *Pseudomonas aeruginosa* ATCC 27853 alone or co-inoculated with the engineered M13-derived phage-display particle P9b or the insert-less vector pC89 (used as a control for phage-related effects not associated with the displayed insert). Data are expressed as percentages derived from three independent biological replicates, while the corresponding absolute values (mean ± SD) are reported in Table S3. Statistical significance was evaluated using one-way ANOVA followed by Tukey’s post hoc test for multiple comparisons (Table S4). Adjusted *p*-values < 0.05, <0.01, and <0.001 are indicated by one (*), two (**), and three (***) asterisks, respectively.

The effect of P9b on biofilm-associated metabolic activity (XTT assay) of P9b was assessed at different stages of biofilm development, namely 24, 48, and 72 h of incubation (Figure 2, Table S3).

At all time analyzed points, P9b induced a marked reduction in biofilm metabolic activity compared with the untreated control. After 24 h of incubation, P9b-treated samples displayed a pronounced decrease in biofilm-associated metabolic activity, with a mean difference of about 55% (*p* < 0.0009). Although the inhibitory effect slightly decreased at 48 h, the reduction remained substantial (mean difference of about 40%; *p* = 0.0011), indicating that P9b continued to impair biofilm development during its maturation phase. At 72 h, a consistent reduction was still observed (mean difference of about 45%; *p* < 0.001), confirming that P9b maintains antibiofilm activity even at advanced stages of biofilm growth, when biofilms are typically denser and metabolically more stable. No significant differences were observed between the untreated control and the sample treated with the insert-less vector pC89 at any time point.

### 3.3. Effect of P9b on P. aeruginosa Pyocyanin Production

**Figure 3:**
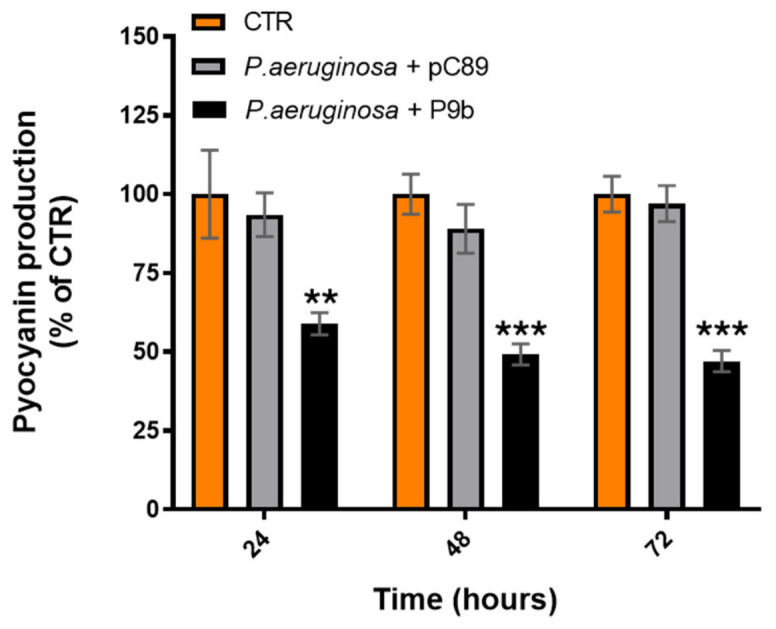
Pyocyanin production (OD_405_) of *Pseudomonas aeruginosa* ATCC 27853 alone or co-inoculated with the engineered M13-derived phage-display particle P9b or the insert-less vector pC89 (used as a control for phage-related effects not associated with the displayed insert). Data are expressed as percentages derived from three independent biological replicates, while the corresponding absolute values (mean ± SD) are reported in Table S5. Statistical significance was evaluated using one-way ANOVA followed by Tukey’s post hoc test for multiple comparisons (Table S6). Adjusted *p*-values < 0.05, <0.01, and <0.001 are indicated by one (*), two (**), and three (***) asterisks, respectively.

To further assess the impact of P9b on virulence-associated traits, pyocyanin production was quantified at the same time points selected for biofilm inhibition analysis (24, 48, and 72 h), allowing direct comparison between antibiofilm and antivirulence effects (Figure 3, Table S4).

After 24 h of incubation, P9b-treated samples showed a reduction in pyocyanin production compared with the untreated control (mean difference of about 41%; *p* = 0.0038). This effect became more pronounced at 48 h (~51%) and was maintained at 72 h (~53%), indicating sustained interference with phenazine production throughout bacterial growth. Consistent with the results observed for growth and biofilm metabolic activity, no significant differences were detected between the untreated control and the insert-less vector pC89 at any time point.

### 3.4. Effect of P9b on QS-Related Gene Expression

**Figure 4:**
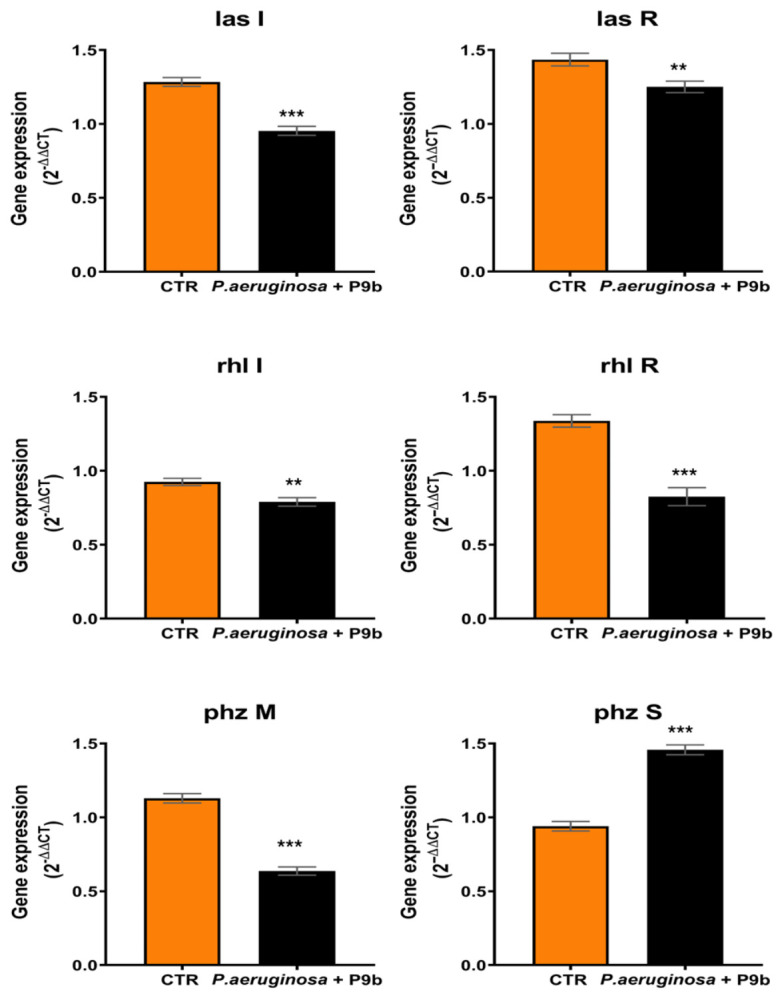
Relative expression of quorum sensing and phenazine genes (*lasI*, *lasR*, *rhlI*, *RhlR*, *phzM*, and *phzS*) in *Pseudomonas aeruginosa* ATCC 27853 alone or co-inoculated with the engineered M13-derived phage-display particle P9b. Data represent the means of three independent replicates. Statistical analysis was performed using Unpaired *t* test (Table S7). Asterisks indicate levels of significance: *p* < 0.05 (*), *p* < 0.01 (**), *p* < 0.001 (***) compared to the CTR.

To explore the molecular basis underlying the observed effects on virulence-associated phenotypes, the expression levels of key quorum sensing (QS) regulators (*lasI*, *lasR*, *rhlI*, and *rhlR*) and phenazine biosynthesis genes (*phzM* and *phzS*) were evaluated following exposure to P9b (Figure 4, Table S5).

Quantitative gene expression analysis suggests a modulation of all analyzed QS regulators in the presence of P9b compared with the untreated control (Figure 4; Table S5). Specifically, *lasI* expression was downregulated (mean difference of about 26%; *p* = 0.0002). Similarly, *lasR* showed downregulation (mean difference of about 13%; *p* = 0.0052). Within the Rhl system, *rhlI* expression was also reduced (mean difference of about 15%; *p* = 0.0034), while *rhlR* exhibited the strongest downregulation among QS regulators (mean difference of about 38%; *p* = 0.0003). Genes involved in phenazine biosynthesis were likewise affected. *phzM* expression was markedly downregulated (mean difference of about 44%; *p* < 0.0001). In contrast, *phzS* was upregulated (mean difference of about 55%; *p* < 0.0001).

### 3.5. Binding Specificity and Activity of P9b Across P. aeruginosa Clinical Isolates

**Figure 5:**
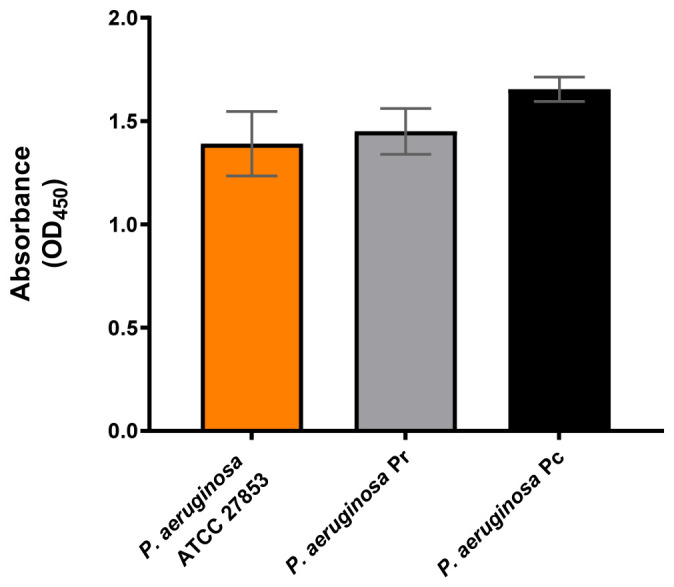
ELISA-based evaluation of engineered M13-derived phage-display particleP9b binding to *Pseudomonas aeruginosa* ATCC 27853 and clinical strain isolates. Data represent the means of three independent replicates. Statistical significance was evaluated using one-way ANOVA followed by Tukey’s post hoc test for multiple comparisons (Table S8).

To evaluate the target specificity of the engineered phage, an ELISA-based binding assay was performed using *P. aeruginosa* ATCC 27853 and multiple clinical isolates (Figure 5).

P9b exhibited strong and specific binding to all tested clinical isolates, with signal intensities comparable to those obtained for the reference strain. In contrast, the insert-less vector pC89 did not show significant binding to either the reference or clinical strains.

**Figure 6:**
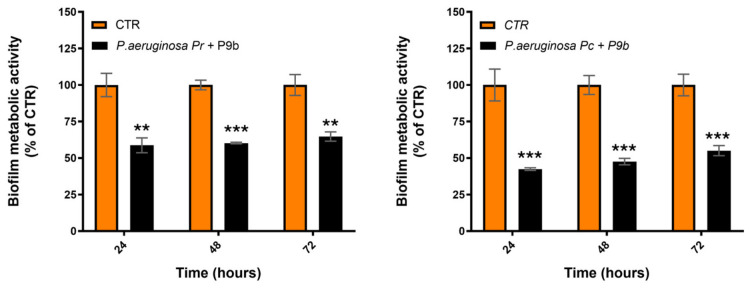
Biofilm metabolic activity (XTT, OD_490_) of *Pseudomonas aeruginosa* clinical strains alone or co-inoculated with the engineered M13-derived phage-display particleP9b. Data are expressed as percentages derived from three independent replicates, while the corresponding absolute values (mean ± SD) are reported in Table S9. Statistical analysis was performed using Unpaired *t* test (Table S10). Asterisks indicate levels of significance: *p* < 0.05 (*), *p* < 0.01 (**), *p* < 0.001 (***).

The antibiofilm activity of P9b was subsequently evaluated against different *P. aeruginosa* clinical strains (Figure 6, Table S6).

For the Pr strain, P9b treatment resulted in a significant reduction in biofilm-associated metabolic activity at all evaluated time points. At 24 h, biofilm production was decreased (mean difference of about 42%; *p* = 0.0016). The inhibitory effect was even more pronounced at 48 h (mean difference of about 39%; *p* < 0.0001). At 72 h, a reduction persisted (mean difference of about 36%; *p* = 0.0016), indicating sustained antibiofilm activity over time. However, the magnitude of inhibition observed in the Pr strain was lower than that detected in the laboratory reference strain *P. aeruginosa* ATCC 27853 at comparable time points.

In the Pc strain, P9b inhibited biofilm-associated metabolic activity at all time points analyzed, as measured by the XTT assay. At 24 h, the reduction was marked (mean difference of about 57%; *p* = 0.0008). Biofilm inhibition was maintained at 48 h (mean difference of about 53%; *p* = 0.0002) and at 72 h (mean difference of about 45%; *p* = 0.0007). Notably, the magnitude of inhibition in the Pc strain was comparable to that observed in the ATCC 27853 reference strain, indicating that P9b retains strong antibiofilm activity in this clinical isolate.

## 4. Discussion

In this study, we investigated the impact of the engineered M13-derived phage-display particle P9b on *Pseudomonas aeruginosa* physiology, integrating growth and virulence-associated phenotypes. Importantly, our findings suggest that the antivirulence activity of P9b is not dependent on phage replication or genetic manipulation, but rather on its ability to bind a bacterial surface target. Consistent with this, P9b appears to act as a selective, non-lytic modulator of virulence-associated pathways, exerting transient effects on planktonic growth and decrease in biofilm metabolic activity and virulence factor production.

Growth curve analyses (Figure 1) showed that P9b induced a transient delay during early exponential growth, manifested as a temporary extension of the lag phase, without affecting overall long-term proliferation. The early and reversible effect could be due to the interaction between the engineered phage particles and the bacterial surface that transiently alters envelope homeostasis and cellular resources required for growth. Binding of the engineered filamentous phage to its bacterial target, previously identified as an outer membrane protein (Omp) [21,23], could potentially be associated with changes in membrane organization or surface properties [31,32]. This mode of interaction differs from that commonly employed by M13 phages during the infection process, which relies on recognition of the F pilus mediated by the minor coat protein pIII [33]. In contrast, target recognition by P9b is driven by a foreign peptide displayed on the major coat protein pVIII, thereby establishing an alternative and non-canonical interaction with the bacterial surface [34,35,36]. Although productive entry is unlikely in this system, binding P9b to its bacterial targets may induce local effects at the cell surface. In Gram-negative bacteria, systems such as Tol-Pal/TolQRA ensure coordination between the inner and outer membranes and contribute to envelope integrity through proton motive force-dependent processes [37,38,39]. In this context, any perturbation of envelope dynamics remains hypothetical and requires further experimental validation. This effect does not appear to influence either the subsequent growth rate or the maximum cell density, suggesting a reversible and non-lethal perturbation. Such behavior may be related to the non-replicative nature of the engineered phage, which could lead to a progressive decrease in the phage-to-cell ratio as the bacterial population expands, thereby attenuating its inhibitory effect over time.

In contrast to the transient effect observed on planktonic growth, P9b exhibited a robust and sustained antibiofilm activity, accompanied by a complex and time-dependent modulation of pyocyanin production. Biofilm inhibition (Figure 2) was more pronounced during the early stages (first 24 h) and remained significant at later incubation time points (48–72 h). Similarly, pyocyanin production (Figure 3) was overall reduced, although the magnitude of the effect appeared to increase over time. The observed physiological response may reflect an underlying regulatory adaptation that can be better interpreted in light of the transcriptional modulation of quorum sensing (QS) regulators and phenazine biosynthesis genes (Figure 4). P9b induced a significant downregulation of the core QS circuitry, including both the Las (*lasI*, *lasR*) and Rhl (*rhlI*, *rhlR*) systems, with the strongest effect observed for *rhlR*. Given the central role of these regulators in coordinating biofilm maturation and structural stability, their repression is consistent with the observed early and pronounced reduction in biofilm-associated metabolic activity. In particular, inhibition of the Las system, which is hierarchically positioned at the top of the QS regulatory network in *P. aeruginosa* [40,41,42], may contribute to alterations in the downstream activation cascade, potentially affecting the coordinated expression of adhesion factors, extracellular matrix components, and other biofilm-associated determinants. The persistence of antibiofilm activity at later time points may indicate that bacterial populations partially compensate for QS disruption over time, possibly through alternative regulatory pathways or adaptive responses [43,44].

In parallel, the expression profile of phenazine biosynthesis genes provides insight into the observed dynamics of pyocyanin production. The strong downregulation of *phzM*, which catalyzes a key step in the conversion of phenazine-1-carboxylic acid (PCA) toward pyocyanin, may be associated with reduced pyocyanin biosynthesis, particularly at early stages [45,46]. However, the concomitant and significant upregulation of *phzS* suggests a shift in phenazine metabolic flux. Since PhzS catalyzes the conversion of intermediates into pyocyanin, its increased expression may progressively compensate for upstream limitations, which could help explain the delayed increase in pyocyanin levels over time [45,46,47]. This decoupling between QS repression and selective activation of downstream phenazine-modifying enzymes points to a more nuanced regulatory rewiring rather than a uniform suppression of virulence pathways. These observations should be interpreted with caution, as the underlying mechanisms were not directly investigated.

Preliminary results indicate that the activity of P9b is not restricted to the laboratory reference strain but is also conserved across clinically relevant *P. aeruginosa* isolates. ELISA-based binding assays (Figure 5) suggest that P9b retains strong and specific affinity toward all tested clinical strains, with signal intensities comparable to those observed for the ATCC 27853 reference strain, while the control phage showed no detectable interaction. These findings indicate that the molecular target recognized by P9b is conserved at the species level, supporting the robustness of the phage display–selected interaction and its potential applicability in heterogeneous clinical contexts. Consistent with this, P9b maintained significant antibiofilm activity across all tested clinical isolates, although with strain-dependent quantitative variability (Figure 6). In the Pr strain, biofilm inhibition was sustained over time but less pronounced compared to the reference strain, suggesting partial resistance or compensatory regulatory mechanisms. In contrast, the Pc isolate exhibited a strong and persistent reduction in biofilm metabolic activity, with inhibition levels comparable to those observed in ATCC 27853. This variability is in line with the well-established heterogeneity of QS hierarchies, regulatory network plasticity, and biofilm-forming capacity among clinical *P. aeruginosa* isolates.

While the results are promising, this study has some limitations. First, all experiments were conducted under in vitro conditions, which may not fully reflect the complexity of in vivo environments. Second, only a limited number of clinical *P. aeruginosa* isolates were included, which may not capture the full heterogeneity of this pathogen. Therefore, further studies involving a broader panel of clinical strains and more physiologically relevant models are needed to confirm and extend these observations.

## 5. Conclusions

Taken together, our findings suggest that the engineered M13-derived phage-display particle P9b functions as a non-lytic antivirulence agent capable of modulating quorum sensing (QS)-dependent regulatory networks in *Pseudomonas aeruginosa*. By selectively interfering with QS circuits through specific surface binding, P9b induces a sustained impairment of biofilm formation and a modulation of virulence-associated phenotypes without exerting bactericidal effects.

Importantly, P9b retained binding capacity and antibiofilm activity across multiple clinical isolates, supporting the targeting of conserved surface-associated determinants beyond the reference strain. However, the observed strain-dependent variability suggests the influence of bacterial heterogeneity and suggests that these findings should be considered preliminary.

Although this study is limited by its in vitro design and the relatively small number of clinical isolates analyzed, it suggests a potential antivirulence mechanism whereby a phage display–derived filamentous phage may influence QS-related phenotypes through specific surface binding, opening new perspectives for non-lytic, target-driven antimicrobial strategies. Further investigations involving a broader panel of strains and more physiologically relevant models are required to validate and extend these findings.

## Data Availability

The original contributions presented in this study are included in the article. Further inquiries can be directed to the corresponding authors.

## Supplementary Materials

The following supporting information can be downloaded at: https://www.mdpi.com/article/10.3390/microorganisms14051028/s1, Figure S1. Growth kinetics (OD540) of *Pseudomonas aeruginosa* ATCC 27853 alone or co-inoculated with the engineered M13-derived phage-display particleP9b or the insert-less vector pC89 (used as a control for phage-related effects not associated with the displayed insert). Statistical significance was evaluated using one-way ANOVA followed by Tukey’s post hoc test for multiple comparisons (Table S2). Adjusted *p*-values < 0.05, <0.01, and <0.001 are indicated by one (*), two (**), and three (***) asterisks, respectively; Table S1. Tukey’s multiple comparisons test for *Pseudomonas aeruginosa* growth (CFU/mL) in the presence or absence of engineered M13-derived phage-display particleP9b or the insert-less vector pC89; Table S2. Tukey’s multiple comparisons test for *Pseudomonas aeruginosa* growth (OD540) in the presence or absence of M13-derived phage-display particleP9b or the insert-less vector pC89; Table S3. Absolute values (OD_490_, mean ± SD) for *Pseudomonas aeruginosa* biofilm (XTT, OD_490_) in the presence or absence of engineered M13-derived phage-display particle P9b or the insert-less vector pC89; Table S4. Tukey’s multiple comparisons test for *Pseudomonas aeruginosa* biofilm (XTT, OD_490_) in the presence or absence of engineered M13-derived phage-display particle P9b or the insert-less vector pC89; Table S5. Absolute values (OD_490_, mean ± SD) for *Pseudomonas aeruginosa* pyocyanin (OD_405_) in the presence or absence of engineered M13-derived phage-display particle P9b or the insert-less vector pC89; Table S6. Tukey’s multiple comparisons test for *Pseudomonas aeruginosa* pyocyanin (OD_405_) in the presence or absence of engineered M13-derived phage-display particleP9b or the insert-less vector pC89; Table S7. Unpaired *t* test for *Pseudomonas aeruginosa* gene expression in the presence or absence of engineered M13-derived phage-display particleP9b; Table S8. Tukey’s multiple comparisons test for ELISA-based evaluation of engineered M13-derived phage-display particle P9b binding to *Pseudomonas aeruginosa* clinical strain isolates; Table S9. Absolute values (OD_490_, mean ± SD) for biofilm (XTT, OD_490_) from *Pseudomonas aeruginosa* clinical strains (*Pr* and *Pc*) in the presence or absence of M13-derived phage-display particle P9b; Table S10. Unpaired *t* test for *Pseudomonas aeruginosa* clinical strains (Pr and Pc) in the presence or absence of engineered M13-derived phage-display particle P9b.
