# Supplementary material for: Engineered Phage Modulates Quorum Sensing and Biofilm Formation in Pseudomonas aeruginosa
Source: Microorganisms. 2026 Apr 30;14(5):1028. doi: 10.3390/microorganisms14051028 (PMC13209296; doi:10.3390/microorganisms14051028)
Supplement: Supplementary file 1 [file microorganisms-14-01028-s001.zip › microorganisms-4260325-supplementary.pdf]

Supplementary information

Attenuation of *Pseudomonas aeruginosa* virulence by engineered phage

Domenico Franco[1,*], Salvatore Papasergi[2], Francesco Mediati[1,3], Salvatore P.P. Guglielmino and Laura Maria De Plano[1,*]

[1] Department of Chemical, Biological, Pharmaceutical and Environmental Sciences (ChiBioFarAm), University of Messina, Viale F. Stagno d'Alcontres 31, 98166 Messina Italy. DF dfranco@unime.it, FM francesco.mediati@studenti.unime.it, SPPG sguglielm@unime.it, LMDP ldeplano@unime.it.

[2] Institute of Translational Pharmacology of the National Research Council, Via Fosso del Cavaliere, 100 (c/o Area di Ricerca di Tor Vergata), 00133 Roma. SP salvatore.papasergi@cnr.it.

[3] Department of Occupational and Environmental Medicine, Epidemiology and Hygiene, Italian Workers' Compensation Authority (INAIL), Contrada Ficarella, 88046 Lamezia Terme, Italy.

* Correspondence: DF dfranco@unime.it; LMDP ldeplano@unime.it

Table S1. Tukey's multiple comparisons test for *Pseudomonas aeruginosa* growth (CFU/mL) in the presence or absence of M13-derived phage-display particle P9b or the insert-less vector pC89

Time (h)	Conditions	Mean Diff,	95,00% CI of diff,	Significant ?	Summary	Adjusted P Value
0	CTR vs. *P. aeruginosa* + P9b	1100000	-5542944 to 7742944	No	ns	0,8703
	CTR vs. *P. aeruginosa* + pC89	1166667	-5476277 to 7809610	No	ns	0,8557
2	CTR vs. *P. aeruginosa* + P9b	8266667	-7302566 to 23835900	No	ns	0,3051
	CTR vs. *P. aeruginosa* + pC89	1333333	-14235900 to 16902566	No	ns	0,9629
4	CTR vs. *P. aeruginosa* + P9b	226666667	143223011 to 310110322	Yes	***	0,0004
	CTR vs. *P. aeruginosa* + pC89	33000000	-50443655 to 116443655	No	ns	0,4885
6	CTR vs. *P. aeruginosa* + P9b	539333333	11605968 to 1067060698	Yes	*	0,0460
	CTR vs. *P. aeruginosa* + pC89	65000000	-462727365 to 592727365	No	ns	0,9252
8	CTR vs. *P. aeruginosa* + P9b	1185000000	-222778248 to 2592778248	No	ns	0,0919
	CTR vs. *P. aeruginosa* + pC89	130000000	-1277778248 to 1537778248	No	ns	0,9570
24	CTR vs. *P. aeruginosa* + P9b	213333333	-4027517570 to 4454184236	No	ns	0,9870
	CTR vs. *P. aeruginosa* + pC89	0,000	-4240850903 to 4240850903	No	ns	>0,9999
48	CTR vs. *P. aeruginosa* + P9b	436666667	-2931289123 to 3804622457	No	ns	0,9176
	CTR vs. *P. aeruginosa* + pC89	113333333	-3254622457 to 3481289123	No	ns	0,9941
72	CTR vs. *P. aeruginosa* + P9b	160000000	-2031377252 to 2351377252	No	ns	0,9728
	CTR vs. *P. aeruginosa* + pC89	-10000000	-2201377252 to 2181377252	No	ns	0,9999

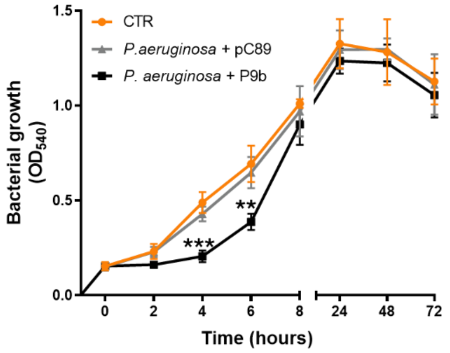

Figure S1. Growth kinetics (OD_{540}) of *Pseudomonas aeruginosa* ATCC 27853 alone or co-inoculated with the phage clone P9b or the insert-less vector pC89 (used as a control for phage-related effects not associated with the displayed insert). Statistical significance was

evaluated using one-way ANOVA followed by Tukey's post hoc test for multiple comparisons (Table S2). Adjusted *p*-values < 0.05, < 0.01, and < 0.001 are indicated by one (*), two (**), and three (***) asterisks, respectively.

Table S2. Tukey's multiple comparisons test for *Pseudomonas aeruginosa* growth (OD_{540}) in the presence or absence of M13-derived phage-display particle P9b or the insert-less vector pC89

Time (h)	Conditions	Mean Diff,	95,00% CI of diff,	Significant ?	Summary	Adjusted P Value
	CTR vs. *P. aeruginosa* + P9b	-0,0003333	-0,04849 to 0,04782	No	ns	0,9998
0	CTR vs. *P. aeruginosa* + pC89	0,002667	-0,04549 to 0,05082	No	ns	0,9843
	CTR vs. *P. aeruginosa* + P9b	0,07033	-0,001948 to 0,1426	No	ns	0,0554
2	CTR vs. *P. aeruginosa* + pC89	0,002000	-0,07028 to 0,07428	No	ns	0,9960
	CTR vs. *P. aeruginosa* + P9b	0,2837	0,1761 to 0,3912	Yes	***	0,0005
4	CTR vs. *P. aeruginosa* + pC89	0,06000	-0,04757 to 0,1676	No	ns	0,2762
	CTR vs. *P. aeruginosa* + P9b	0,3057	0,1138 to 0,4975	Yes	**	0,0066
6	CTR vs. *P. aeruginosa* + pC89	0,04600	-0,1458 to 0,2378	No	ns	0,7527
	CTR vs. *P. aeruginosa* + P9b	0,1100	-0,1396 to 0,3596	No	ns	0,4205
8	CTR vs. *P. aeruginosa* + pC89	0,04067	-0,2089 to 0,2903	No	ns	0,8740
	CTR vs. *P. aeruginosa* + P9b	0,09167	-0,1656 to 0,3490	No	ns	0,5521
24	CTR vs. *P. aeruginosa* + pC89	0,03200	-0,2253 to 0,2893	No	ns	0,9239
	CTR vs. *P. aeruginosa* + P9b	0,05733	-0,2424 to 0,3571	No	ns	0,8320
48	CTR vs. *P. aeruginosa* + pC89	-0,01467	-0,3144 to 0,2851	No	ns	0,9877
	CTR vs. *P. aeruginosa* + P9b	0,07200	-0,2631 to 0,4071	No	ns	0,7943
72	CTR vs. *P. aeruginosa* + pC89	0,01633	-0,3187 to 0,3514	No	ns	0,9878

Table S3. Absolute values (OD_{490}, mean ± SD) for *Pseudomonas aeruginosa* biofilm (XTT, OD_{490}) in the presence or absence of M13-derived phage-display particle P9b or the insert-less vector pC89

Time (h)	Conditions		
	CTR	*P. aeruginosa* + P9b	*P. aeruginosa* + pC89
24	1.56 ± 0.16	0.69 ± 0.18	1.57 ± 0.10
48	1.25 ± 0.09	0.74 ± 0.01	1.22 ± 0.13
72	1.28 ± 0.13	0.70 ± 0.06	1.21 ± 0.02

Table S4. Tukey's multiple comparisons test for *Pseudomonas aeruginosa* biofilm (XTT, OD_{490}) in the presence or absence of M13-derived phage-display particle P9b or the insert-less vector pC89

Time (h)	Conditions	Mean Diff,	95,00% CI of diff,	Significant ?	Summary	Adjusted P Value
	CTR vs. *P. aeruginosa* + P9b	0,8613	0,4939 to 1,229	Yes	***	0,0009
24	CTR vs. *P. aeruginosa* + pC89	-0,01163	-0,3791 to 0,3558	No	ns	0,9948
	CTR vs. *P. aeruginosa* + P9b	0,5165	0,2867 to 0,7462	Yes	**	0,0011
48	CTR vs. *P. aeruginosa* + pC89	0,03062	-0,1992 to 0,2604	No	ns	0,9133
	CTR vs. *P. aeruginosa* + P9b	0,5761	0,3618 to 0,7904	Yes	***	0,0004
72	CTR vs. *P. aeruginosa* + pC89	0,06647	-0,1478 to 0,2807	No	ns	0,6306

Table S5. Absolute values (OD_{490}, mean ± SD) for *Pseudomonas aeruginosa* pyocyanin (OD_{405}) in the presence or absence of M13-derived phage-display particle P9b or the insert-less vector pC89

Time (h)	Conditions		
	CTR	*P. aeruginosa* + P9b	*P. aeruginosa* + pC89
24	0.79 ± 0.11	0.46 ± 0.03	0.74 ± 0.05
48	0.87 ± 0.06	0.43 ± 0.03	0.78 ± 0.07
72	0.94 ± 0.05	0.44 ± 0.02	0.91 0.05

Table S6. Tukey's multiple comparisons test for *Pseudomonas aeruginosa* pyocyanin (OD_{405}) in the presence or absence of M13-derived phage-display particle P9b or the insert-less vector pC89

Time (h)	Conditions	Mean Diff,	95,00% CI of diff,	Significant ?	Summary	Adjusted P Value
24	CTR vs. *P. aeruginosa* + P9b	0,3236	0,1418 to 0,5054	Yes	**	0,0038
	CTR vs. *P. aeruginosa* + pC89	0,05154	-0,1302 to 0,2333	No	ns	0,6770
48	CTR vs. *P. aeruginosa* + P9b	0,4428	0,3098 to 0,5757	Yes	***	0,0001
	CTR vs. *P. aeruginosa* + pC89	0,09610	-0,03687 to 0,2291	No	ns	0,1464
72	CTR vs. *P. aeruginosa* + P9b	0,4977	0,3780 to 0,6174	Yes	***	<0,0001
	CTR vs. *P. aeruginosa* + pC89	0,02839	-0,09132 to 0,1481	No	ns	0,7572

Table S7. Unpaired t test for *Pseudomonas aeruginosa* gene expression in the presence or absence of M13-derived phage-display particle P9b

Gene	Conditions	Mean Diff,	95,00% CI of diff,	Significant ?	Summary	Adjusted P Value
las I	CTR vs. *P. aeruginosa* + P9b	-0,331	-0,399 to -0,263	Yes	***	0,0002
las R	CTR vs. *P. aeruginosa* + P9b	-0,185	-0,277 to -0,0920	Yes	**	0,0052
rhl I	CTR vs. *P. aeruginosa* + P9b	-0,135	-0,196 to -0,0746	Yes	**	0,0034
rhl R	CTR vs. *P. aeruginosa* + P9b	-0,512	-0,631 to -0,393	Yes	***	0,0003
phz M	CTR vs. *P. aeruginosa* + P9b	-0,492	-0,560 to -0,425	Yes	***	<0,0001
phz S	CTR vs. *P. aeruginosa* + P9b	0,517	0,443 to 0,591	Yes	***	<0,0001

Table S8. Tukey's multiple comparisons test for ELISA-based evaluation of M13-derived phage-display particle P9b binding to *Pseudomonas aeruginosa* clinical strain isolates

Conditions	Mean Diff,	95,00% CI of diff,	Significant ?	Summary	Adjusted P Value
***P. aeruginosa* ATCC 27853** vs. ***P. aeruginosa* Pr**	-0,05967	-0,3484 to 0,2291	No	ns	0,8076
***P. aeruginosa* ATCC 27853** vs. ***P. aeruginosa* Pc**	-0,2637	-0,5524 to 0,02507	No	ns	0,0696
***P. aeruginosa* Pr** vs. ***P. aeruginosa* Pc**	-0,2040	-0,4927 to 0,08473	No	ns	0,1559

Table S9. Absolute values (OD_{490}, mean ± SD) for biofilm (XTT, OD_{490}) from *Pseudomonas aeruginosa* clinical strains (*Pr* and *Pc*) in the presence or absence of M13-derived phage-display particle P9b

Time (h)	Conditions			
	CTR	***P. aeruginosa Pr* + P9b**	CTR	***P. aeruginosa Pc*+ P9b**
24	1.39 ± 0.11	0..81 ± 0.07	1.76 ± 0.19	0.75 ± 0.02
48	1.7 ± 0.06	1.03 ± 0.01	1.39± 0.09	0.66 ± 0.08
72	1.99 ± 0.15	1.28. ± 0.06	1.11 ± 0.08	0.61 ± 0.04

Table S10. Unpaired t test for biofilm (XTT, OD_{490}) from *Pseudomonas aeruginosa* clinical strains (*Pr* and *Pc*) in the presence or absence of M13-derived phage-display particle P9b

Time (h)	Conditions	Mean Diff,	95,00% CI of diff,	Significant ?	Summary	Adjusted P Value
24	CTR vs. *P. aeruginosa Pr* + P9b	-0,573	-0,783 to -0,364	Yes	**	0,0016
48	CTR vs. *P. aeruginosa Pr* + P9b	-0,677	-0,771 to -0,582	Yes	***	<0,0001
72	CTR vs. *P. aeruginosa Pr* + P9b	-0,703	-0,958 to -0,449	Yes	**	0,0016
24	CTR vs. *P. aeruginosa Pc* + P9b	-1,01	-1,32 to -0,703	Yes	***	0,0008
48	CTR vs. *P. aeruginosa Pc* + P9b	-0,703	-0,885 to -0,575	Yes	***	0,0002
72	CTR vs. *P. aeruginosa Pc* + P9b	-0,185	-0,277 to -0,0920	Yes	***	0,0007
